# Pulmonary Endarterectomy in Latvia: A National Experience

**DOI:** 10.3390/medicina55010018

**Published:** 2019-01-15

**Authors:** Matiss Sablinskis, Kristaps Sablinskis, Andris Skride

**Affiliations:** 1 Riga Stradins University, 16 Dzirciema str., LV-1007 Riga, Latvia; matiss.sablinskis@gmail.com (M.S.); kristaps.sablinskis@gmail.com (K.S.); 2 Department of Cardiology, Pauls Stradins Clinical University Hospital, 13 Pilsonu str., LV-1012 Riga, Latvia

**Keywords:** chronic thromboembolic pulmonary hypertension, pulmonary hypertension, pulmonary hypertension

## Abstract

*Background and objectives:* Chronic thromboembolic pulmonary hypertension (CTEPH) is a hemodynamic state characterized by chronic obstruction in pulmonary circulation. The treatment of choice is pulmonary endarterectomy (PEA). The aim of our study was to compile and analyze the data of a small, national center, which has not yet been done in the Baltic states. *Materials and methods:* The data of Latvian CTEPH registry in timeframe from 1 September 2007 to 31 December 2016 was retrospectively analyzed and all patients who underwent PEA were included. *Results:* PEA was done for 7 patients. The in-hospital mortality was 14%. The 3-year survival rate was 86%. The procedure restored pulmonary blood pressure to normal values for three of the patients (42%). The remaining four patients (57%) had persistent pulmonary hypertension (mPAP > 30 mmHg), which required continuous therapy. There was a comparable decline in mean mPAP compared to baseline, 53.4 ± 14.4 mmHg to 44.3 ± 30 mmHg, respectively. At 12-month follow-up, there was a significant improvement in functional capacity, as seen by increased 6-min walk test distance and shifts in New York Heart Association functional class. *Conclusions:* Only 16% of all prevalent Latvian CTEPH patients have underwent PEA in the course of nine years, despite it being the treatment of choice for CTEPH. As PEA and other emerging treatment options, such as balloon pulmonary angioplasty, can only be done in expert centers, numerous organizational, logistical, and economic issues arise for patients of smaller countries, where such centers have not yet been created due to lack of experience and limited amount of patients.

## 1. Introduction

Chronic thromboembolic pulmonary hypertension (CTEPH) is classified as group 4 pulmonary hypertension. It is a hemodynamic state characterized by chronic obstruction in pulmonary circulation, which develops when unresolved emboli in pulmonary arteries undergo fibrotic transformation. The associated remodeling and dysfunction of the pulmonary microvasculature are believed to be significant factors contributing to pathology of CTEPH [1,2].

The incidence of CTEPH after acute pulmonary embolism (PE) is approximately 1.5%, but not all CTEPH patients have a history of PE. For this reason routine screening for CTEPH in patients with previous PE is not recommended [1,3]. The signs and symptoms are mostly non-specific in the first stages of the disease, and early diagnosis remains a challenge [1].

The diagnosis can be made on findings that are obtained after more than 3 months of effective anticoagulation to rule out possible subacute pulmonary embolism. CTEPH is a type of pre-capillary pulmonary hypertension, with mPAP ≥25 mmHg and pulmonary artery wedge pressure (PAWP) of ≤15 mmHg assessed by right heart catheterization in the presence of specific diagnostic signs seen by CT angiography and MR imaging, such as ring-like stenoses and chronic total occlusions [1].

The treatment of CTEPH is distinctively unique from other types of pulmonary hypertension. The treatment of choice is pulmonary endarterectomy (PEA) for all patients who are considered operable by an experienced multidisciplinary CTEPH team [1,3]. For the patient to be eligible for the surgery, a sufficient amount of surgically accessible thromboembolic material is required, with a proportional PVR indicating the absence of extensive secondary vasculopathy [3]. The estimated amount of patients who are operable, ranges from 50% to 70% [4].

There are multiple factors contributing to the complexity of the procedure, the localization of the thrombi being one of them. Thromboembolic disease that is located distally from mid-segmental and subsegmental branches is more challenging for the operating surgeon. This is why personnel experience is one of the key points in preoperative assessment and second opinion is often encouraged [5].

A successful procedure provides both significant hemodynamical and clinical improvement for nearly all patients [6]. Pulmonary blood pressure is restored to normal in about 50% of all patients [7]. For others slight residual pulmonary hypertension remains present [8,9]. In 20% of the cases, the residual pulmonary hypertension is symptomatic and requires treatment [8]. To this date the only approved medical therapy for CTEPH is riociguat—soluble adenylate cyclase inhibitor [1].

In the last few years, due to technological advances pulmonary balloon arterioplasty (PBA) has become more widely used for patients who are ineligible for PEA or who have residual pulmonary hypertension after the procedure [10].

PBA should also be performed only in expert centers, as even though the procedure is minimally invasive, it is highly complex and has some risks [11].

There have been many reports on PEA outcomes from large centers, but the aim of our study was to compile and analyze the data of a small, national center, which has not yet been done in the Baltic states.

## 2. Materials and Methods

This is a prospective, observational, single-center study of the Latvian CTEPH registry. We analyzed the registry data in the timeframe between 1 September 2007 and 31 December 2016 and all of the patients who underwent pulmonary endarterectomy were included. This research was approved by Clinical Research Ethics Committee of Pauls Stradins Clinical University hospital Nr. 151209-6L, 15 December 2009.

Diagnosis of emboli in pulmonary arteries had been established by a contrast enhanced pulmonary angiography. Transthoracic echocardiography was done to measure the ejection fraction and tricuspid annular peak systolic excursion. 

Chronic thromboembolic pulmonary hypertension was confirmed by right-heart catheterization with measurements of mPAP, PVR, PAWP, right atrial pressure, cardiac output, and cardiac index. 

The patients were classified in accordance to the New York Health Association (NYHA) functional class and assessed by 6-min walk test (6MWT).

If possible, a follow-up evaluation which consisted of RHC, TTE, and 6MWT was done at 12 months after the surgery to reassess the state of disease and to evaluate patient’s functional state.

One sample Kolmogorov-Smirnov test was used to assess data distribution. The data displaying normal distribution is presented as mean ± standard deviation, and as median (range) for data distributed non-normally.

Significance of difference for parameters displaying normal distribution was tested with paired Student’s *t*-test, and Wilcoxon signed rank test was used for parameters displaying non-normal distribution. Statistical analysis was performed using SPSS version 23 software (SPSS Inc., Chicago, IL, USA) with *p* values less than 0.05 considered significant.

## 3. Results

Pulmonary endarterectomy was done for 7 (16%) of the 44 prevalent patients with CTEPH. For three of them, the procedure was done in Latvia with visiting experts from Poland, but the rest were operated on at the PEA excellence center in Vienna General Hospital. 

The preoperative characteristics of the patients can be seen in Table 1. 

Four of the patients (57%) were men. The mean age at the time of surgery in this small group of patients was 45 ± 15 years and ranged from 31 to 67. The mean time from diagnosis and the date of PEA was relatively long, 704 ± 560 days, respectively. Five of the patients (71%) had a history of deep vein thrombosis. One of the patients was using riociguat (14%), and sildenafil was used by four (57%) other patients. All of the patients were on warfarin.

At the time of diagnosis, all of the patients presented in NYHA functional class III, and the mean 6MWT distance was 268.6 ± 46.2 m.

The procedure was successful for all of the patients. The mean stay in intensive care unit was 11.1 ± 5.6 days. In-hospital mortality after PEA was 14%. Only one of the patients has died in the perioperative period up to this date. For this patient, there was no significant reduction in mPAP, and bilateral lung transplantation was done shortly after, which resulted in diffuse internal bleeding. The patient died from multiple organ failure 15 days after the PEA.

Six of the patients (86%) had a comprehensive follow-up evaluation 12 months after PEA. The postoperative results can be seen in Table 2. Mean mPAP and RAP decreased from 53.4 ± 14.4 and 14 (3–23) mmHg, to 44.3 ± 30 (*p* > 0.05), and 11 (5–27) mmHg (*p* > 0.05), respectively. Mean preoperative PAWP value was 11.6 ± 4 mmHg (*p* > 0.05) with mean PVR of 8.2 ± 2.4 Wood units. After the surgery the mean PAWP had increased to 12.7 ± 10 (*p* > 0.05), however PVR was reduced to 6.8 ± 5.8 Wood units (*p* > 0.05). There were no significant changes in cardiac output and cardiac index values, as they were 4.8 ± 0.4 L/min and 2.4 ± 0.4 L/min/m^2^, post-surgery, respectively.

The mean 6MWD at the time of follow-up was significantly improved at 421.8 ± 113.3 meters (*p* < 0.05 compared to baseline). Two of the patients (29%) improved their NYHA functional class from III to II, and two (29%) were in NYHA class I.

There was a significant reduction in BNP, the mean value decreased from 524.5 ± 88.3 pg/mL to 181.3 ± 101.3 pg/mL (*p* < 0.05).

## 4. Discussion

The calculated incidence of CTEPH in Latvia in 2016 was 5.1 cases per million inhabitants, whereas the prevalence was 15.7 cases per million inhabitants, and it is one of the highest in Europe [12]. The awareness of this disease has markedly increased due to successful informative campaigning for Latvian healthcare professionals. However, the survival rate for patients with CTEPH at one, three, and five years was only 83.8%, 59.0%, and 44.2%, respectively. The one-year survival rate is the lowest amongst other European adult PH registries. 

This study shows, that merely 16% of all prevalent Latvian CTEPH patients have underwent PEA in the course of nine years, despite it being the treatment of choice for CTEPH, which can provide instant improvement of symptoms, or, for some patients, restore normal hemodynamics in pulmonary circulation.

As PEA and other emerging treatment options, such as BPA, can only be done in expert centers, numerous organizational, logistical, and economic issues arise for patients of smaller countries, where such centers have not yet been created due to lack of experience and limited amount of patients. For this reason, only a small number of patients, who are potentially eligible for the surgery, undergo it, possibly explaining the low survival rates for patients with CTEPH in Latvia, as well as the prolonged time from diagnosis to the procedure.

The first surgeries of the three patients were done in Latvia with the assistance of visiting experts from Poland, however due to the small number of patients in need of this procedure every year, we concluded, that it is less expensive and safer to transport the patients who are eligible and can afford to cover the expenses, to an expert center in Vienna.

Elevated pressure in the right heart (mPAP, rAP), increased vascular resistance (high PVR), and diminished heart functions (low CO, CI), coupled with low functional capacity (low 6MWD, NYHA class III) indicate already advanced disease at the time of diagnosis. 

Pulmonary endarterectomy is relatively safe surgical procedure, when performed in expert centers, with reported overall mortality rate of no more than 5% [13]. It was 14% in our study and the 3-year survival rate was 86%, as all of the patients who had their follow-up visit, are still alive.

The procedure restored pulmonary blood pressure to normal values for 3 of our patients (42%). The remaining four (57%) had persistent pulmonary hypertension (mPAP > 30 mmHg), which was symptomatic and required continuous medical therapy with phosphodiesterase-5 inhibitors (PD5-i) (29%) or combination therapy with PD-5i and endothelin receptor antagonists (14%), as riociguat is yet to be state compensated in Latvia.

Evidence shows, that approximately one third of patients have persistent PH after an apparently successful surgery [8]. It may be caused by concomitant small-vessel arteriopathy, which can be troublesome to determine prior the surgery [14].

Recurrent PH is less common and is associated with a further thromboembolic episode after a successful PEA clearance and a confirmed reduction in PH post-PEA [6]. There is still no consensus on definitions of either residual or recurrent PH, as well as the overall long-term outcome for these patients.

The procedure improved functional capacity for four of the patients, as indicated by the marked increase 6-min walk test distance, and shifts in NYHA functional class. These improvements, to a lesser extent, were also seen in patients with persistent PH, confirming the overall positive impact of PEA.

The results show a significant decline in mean BNP level. High BNP levels correlate with right ventricular remodeling and this value can be used as an indicator of right ventricular dysfunction. BNP level of >167.8 pg/mL has also shown to be an independent predictor for worse outcomes in PEA, associated with higher risk for mortality and residual PH [15]. All of the patients in this study had baseline values much higher than that. 

We acknowledge that the data is limited by the small population size and the retrospective nature of this study. Statistically significant difference between was found only for 6MWT distance, NYHA functional class, and BNP values. The only objective parameter to quantify the impact of surgery was 6MWT distance. A questionnaire like SF-36 could have been used to measure overall improvement in quality of life.

## 5. Conclusions

Only 16% of all prevalent Latvian CTEPH patients have underwent PEA in the course of nine years, despite it being the treatment of choice for CTEPH. As PEA and other emerging treatment options, such as balloon pulmonary angioplasty, can only be done in expert centers, numerous organizational, logistical, and economic issues arise for patients of smaller countries, where such centers have not yet been created due to lack of experience and limited amount of patients.

## Figures and Tables

**Table 1:** Preoperative and postoperative characteristics of the patients.

Characteristic	At Baseline	12 Months Postoperatively	*p*
Male, *n* (%)	4 (57)	3 (43)	NA
Female, *n* (%)	3 (43)	3 (43)	NA
Age, mean ± SD, years	45 ± 15	47.3 ± 15	NA
BSA, mean ± SD, m^2^	2.07 ± 0.23	2.04 ± 0.24	NA
Deep venous thrombosis, *n* (%)	5 (71)	4 (57)	NA
6-minute walking test, mean ± SD, m	241.3 ± 49.7	421.8 ± 113.3	0.016
NYHA Class I, *n* (%)	0 (0)	2 (29)	NA
NYHA Class II, *n* (%)	0 (0)	2 (29)	NA
NYHA Class III, *n* (%)	7 (100)	2 (29)	NA
FEV1, mean ± SD, % predicted	59.1 ± 22.4	65.1 ± 19.5	NA
FEV1/FVC, mean ± SD, % predicted	87.4 ± 16.2	89.5 ± 14.9	NA
mPAP, mean ± SD, mmHg	53.4 ± 14.4	44.3 ± 30	0.422
rAP, median (range), mmHg	14 (3–23)	11 (5–27)	0.875
PCWP, mean ± SD, mmHg	11.6 ± 4	12.7 ± 10	0.581
PVR, mean ± SD, wood units	8.2 ± 4	6.8 ± 5.8	0.305
CO, mean ± SD, L/min	4.9 ± 0.3	4.8 ± 0.4	0.412
CI, mean ± SD, L/min/m^2^	2.2 ± 0.4	2.4 ± 0.4	0.23
BNP, mean ± SD, pg/mL	524.5 ± 88.3	181.3 ± 101.3	0.001
TAPSE, mean ± SD, cm	1.8 ± 0.5	1.5 ± 0.4	NA
Ejection fraction, mean ± SD, %	56 ± 2.4	58 ± 1.7	NA

BSA—body surface area; FEV1—forced expiratory volume on first second; FVC—forced vital capacity; mPAP—mean pulmonary artery pressure; rAP—right atrial pressure; PCWP—pulmonary capillary wedge pressure; PVR—pulmonary vascular resistance; CO—cardiac output; CI—cardiac index; NYHA—New York Heart Association; BNP—brain natriuretic peptide; TAPSE—tricuspid annular plane systolic excursion; NA—not applicable; the Wilcoxon signed rank test was used for testing the difference between baseline and postoperative right atrial pressure value, the paired sample *t*-test was used to test the difference between all other values.

**Table 2:** Preoperative and postoperative hemodynamic and functional characteristics of individual patients.

	Preoperatively					12 Months After PEA				
Patient nr.	mPAP, mmHg	PVR, WU	CI, L/min/m^2^	6MWT, m	NYHA f.c.	mPAP, mmHg	PVR, WU	CI, L/min/m^2^	6MWT, m	NYHA f.c.
1	54	8	2.3	340	III	22	4	2.22	510	I
2	45	5.5	2.67	290	III	61	7	1.85	240	III
3	64	11	2.2	263	III	20	2	2.75	450	II
4	76	NA	1.95	230	III	94	18	2.18	500	II
5	54	10.6	2.2	220	III	NA	NA	NA	NA	NA
6	51	8.2	1.91	246	III	50	7	2.14	230	III
7	30	2.4	2.89	325	III	19	3	2.99	503	I

mPAP—mean pulmonary artery pressure; PVR—pulmonary vascular resistance; CI—cardiac index; 6MWT—six minute walk test; NYHANew York Heart Association; NA—not available
